# Supplementary material for: IL-17B/RB Activation in Pancreatic Stellate Cells Promotes Pancreatic Cancer Metabolism and Growth
Source: Cancers (Basel). 2021 Oct 24;13(21):5338. doi: 10.3390/cancers13215338 (PMC8611647; doi:10.3390/cancers13215338)
Supplement: Supplementary file 1 [file cancers-13-05338-s001.zip › cancers-1330593 Supplementary Figure S2.pdf]

A

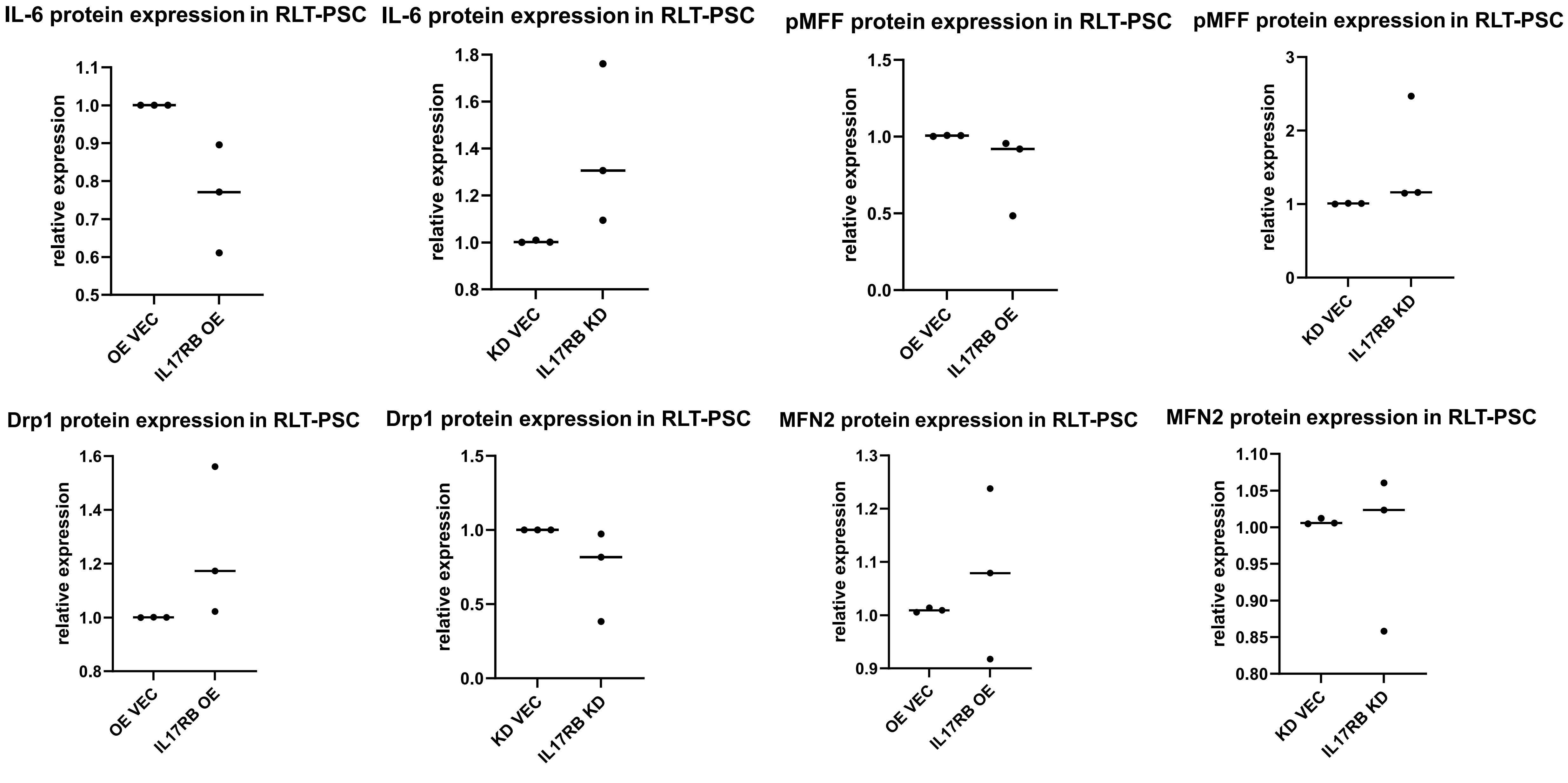
IL-6 protein expression in RLT-PSC
relative expression
1.1
1.0
0.9
0.8
0.7
0.6
0.5
OE VEC
IL17RB OE
IL-6 protein expression in RLT-PSC
relative expression
1.8
1.6
1.4
1.2
1.0
0.8
KD VEC
IL17RB KD
pMFF protein expression in RLT-PSC
relative expression
1.5
1.0
0.5
0.0
OE VEC
IL17RB OE
pMFF protein expression in RLT-PSC
relative expression
3
2
1
0
KD VEC
IL17RB KD
Drp1 protein expression in RLT-PSC
relative expression
1.6
1.4
1.2
1.0
0.8
OE VEC
IL17RB OE
Drp1 protein expression in RLT-PSC
relative expression
1.5
1.0
0.5
0.0
KD VEC
IL17RB KD
MFN2 protein expression in RLT-PSC
relative expression
1.3
1.2
1.1
1.0
0.9
OE VEC
IL17RB OE
MFN2 protein expression in RLT-PSC
relative expression
1.10
1.05
1.00
0.95
0.90
0.85
0.80
KD VEC
IL17RB KD

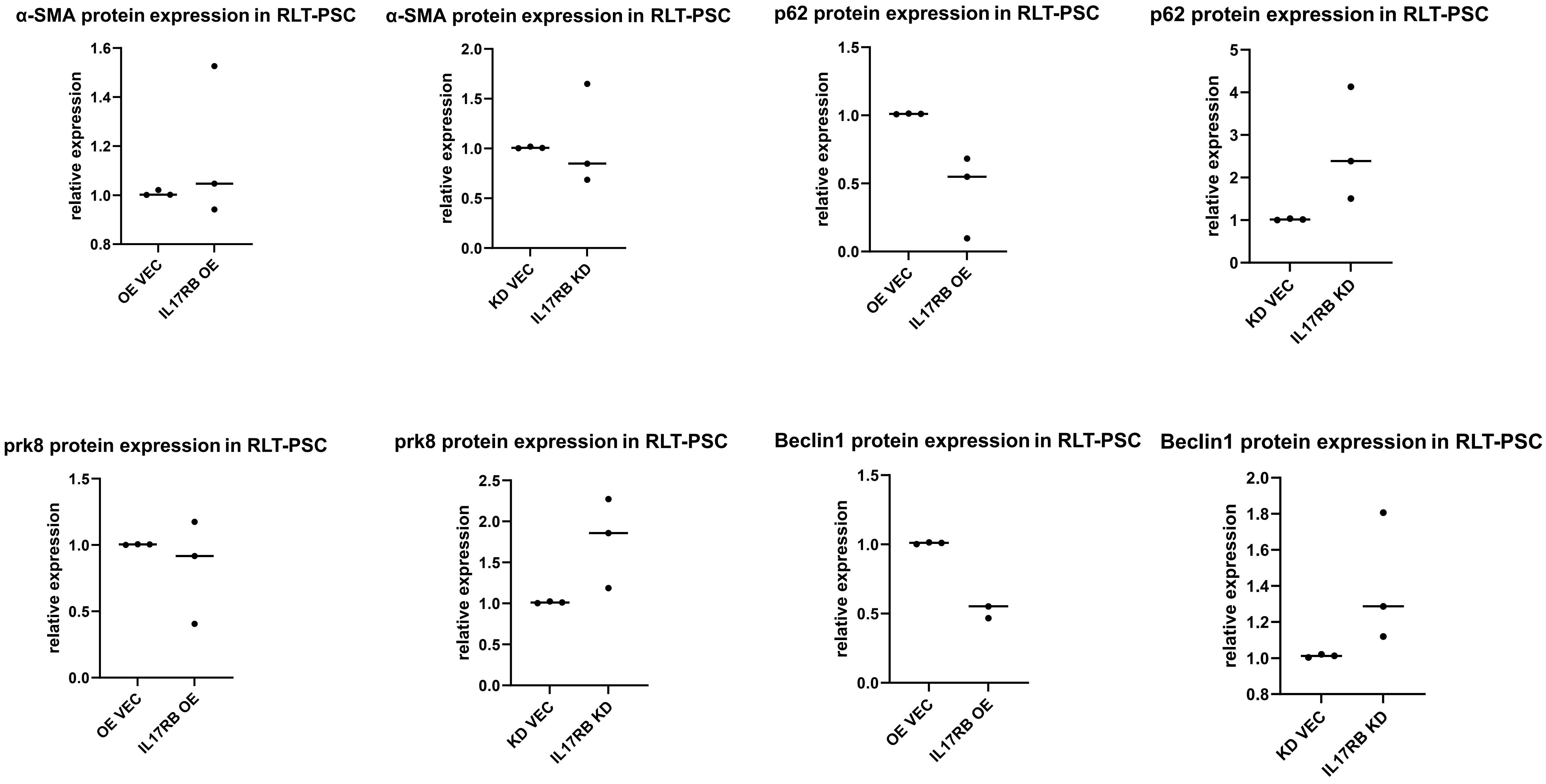
α-SMA protein expression in RLT-PSC
relative expression
1.6
1.4
1.2
1.0
0.8
OE VEC
IL17RB OE
α-SMA protein expression in RLT-PSC
relative expression
2.0
1.5
1.0
0.5
0.0
KD VEC
IL17RB KD
p62 protein expression in RLT-PSC
relative expression
1.5
1.0
0.5
0.0
OE VEC
IL17RB OE
p62 protein expression in RLT-PSC
relative expression
5
4
3
2
1
0
KD VEC
IL17RB KD
prk8 protein expression in RLT-PSC
relative expression
1.5
1.0
0.5
0.0
OE VEC
IL17RB OE
prk8 protein expression in RLT-PSC
relative expression
2.5
2.0
1.5
1.0
0.5
0.0
KD VEC
IL17RB KD
Beclin1 protein expression in RLT-PSC
relative expression
1.5
1.0
0.5
0.0
OE VEC
IL17RB OE
Beclin1 protein expression in RLT-PSC
relative expression
2.0
1.8
1.6
1.4
1.2
1.0
0.8
KD VEC
IL17RB KD

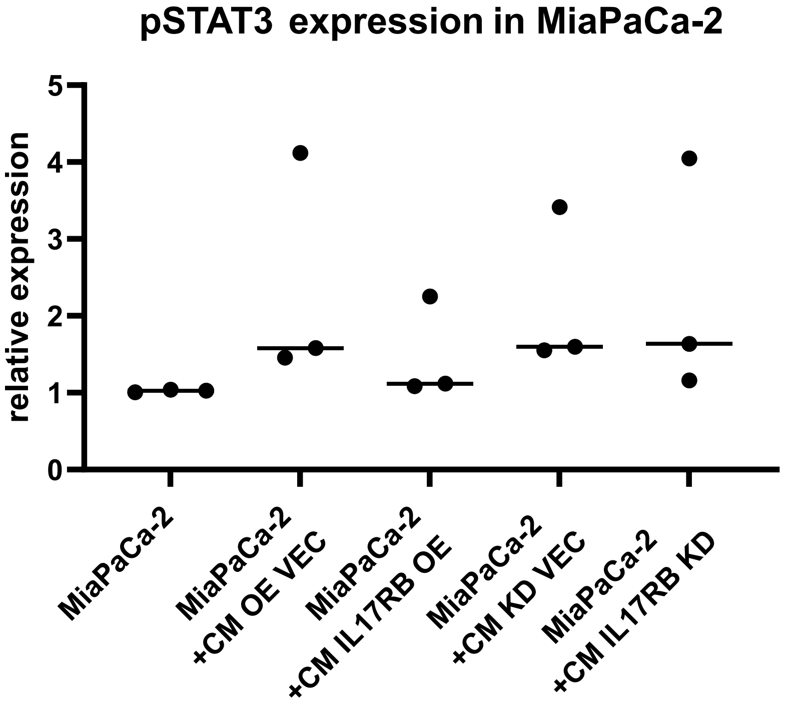
pSTAT3 expression in MiaPaCa-2
relative expression
0
1
2
3
4
5
MiaPaCa-2
MiaPaCa-2 +CM OE VEC
MiaPaCa-2 +CM IL17RB OE
MiaPaCa-2 +CM KD VEC
MiaPaCa-2 +CM IL17RB KD

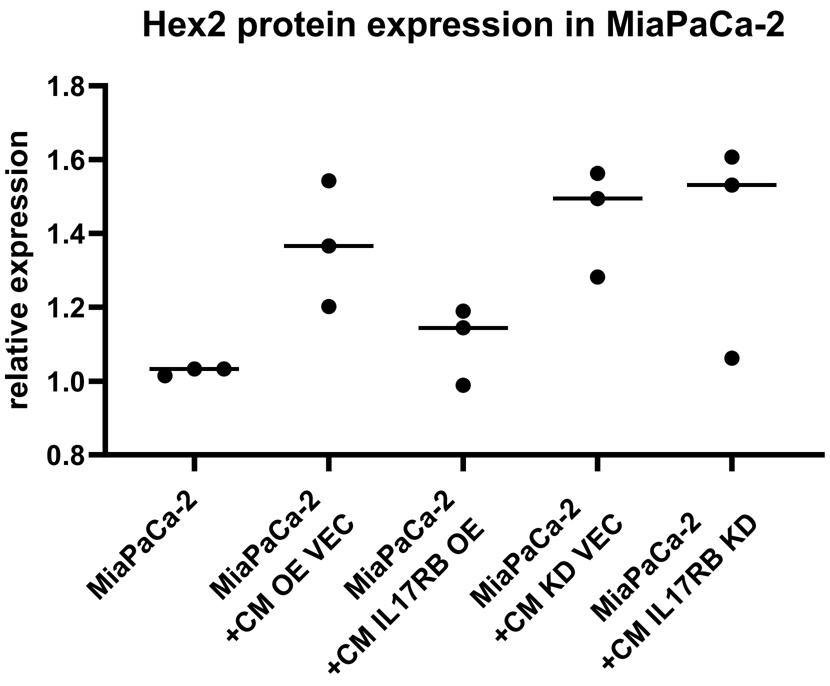
Hex2 protein expression in MiaPaCa-2
relative expression
0.8
1.0
1.2
1.4
1.6
1.8
MiaPaCa-2
MiaPaCa-2 +CM OE VEC
MiaPaCa-2 +CM IL17RB OE
MiaPaCa-2 +CM KD VEC
MiaPaCa-2 +CM IL17RB KD

Original Western Blot data

B

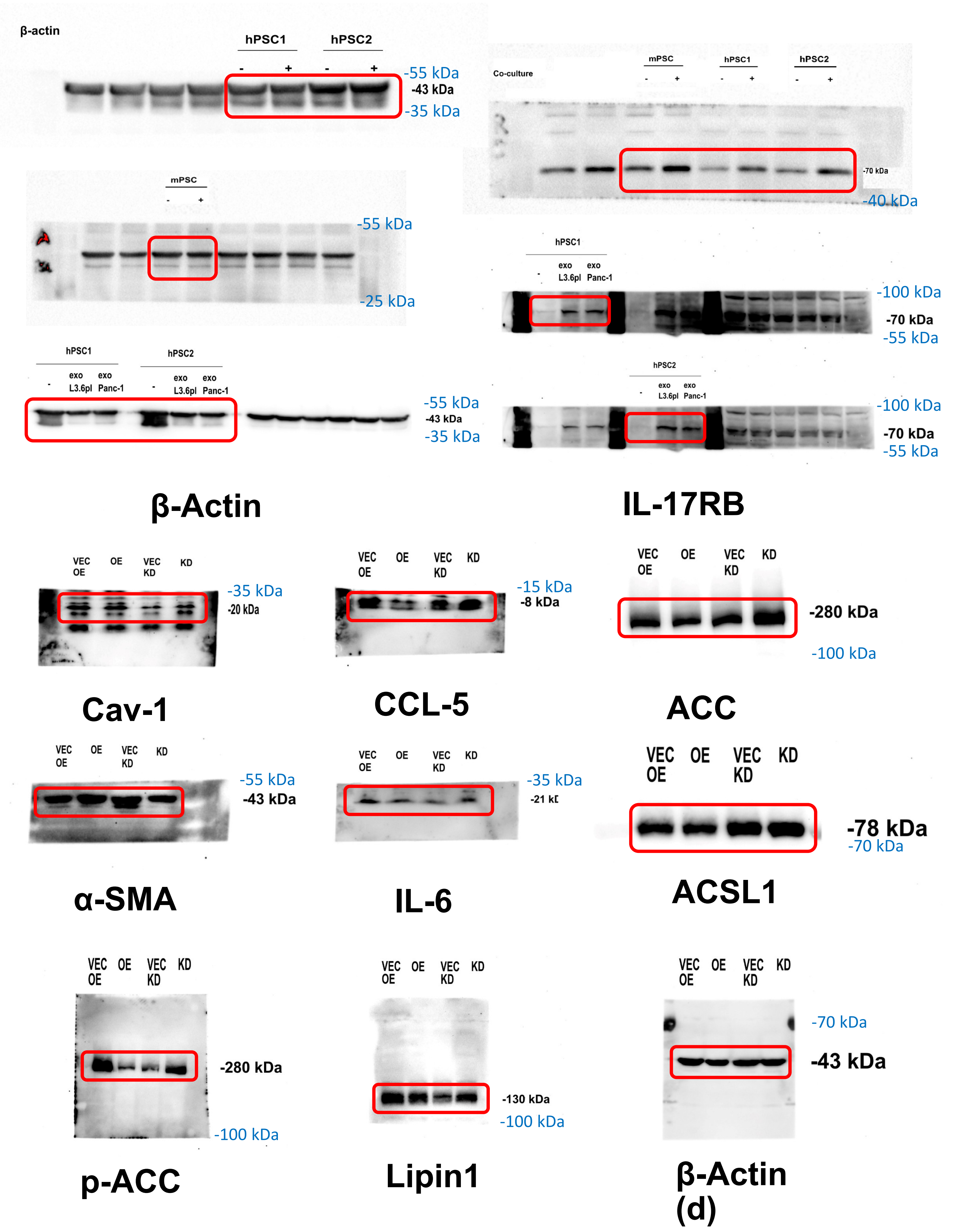

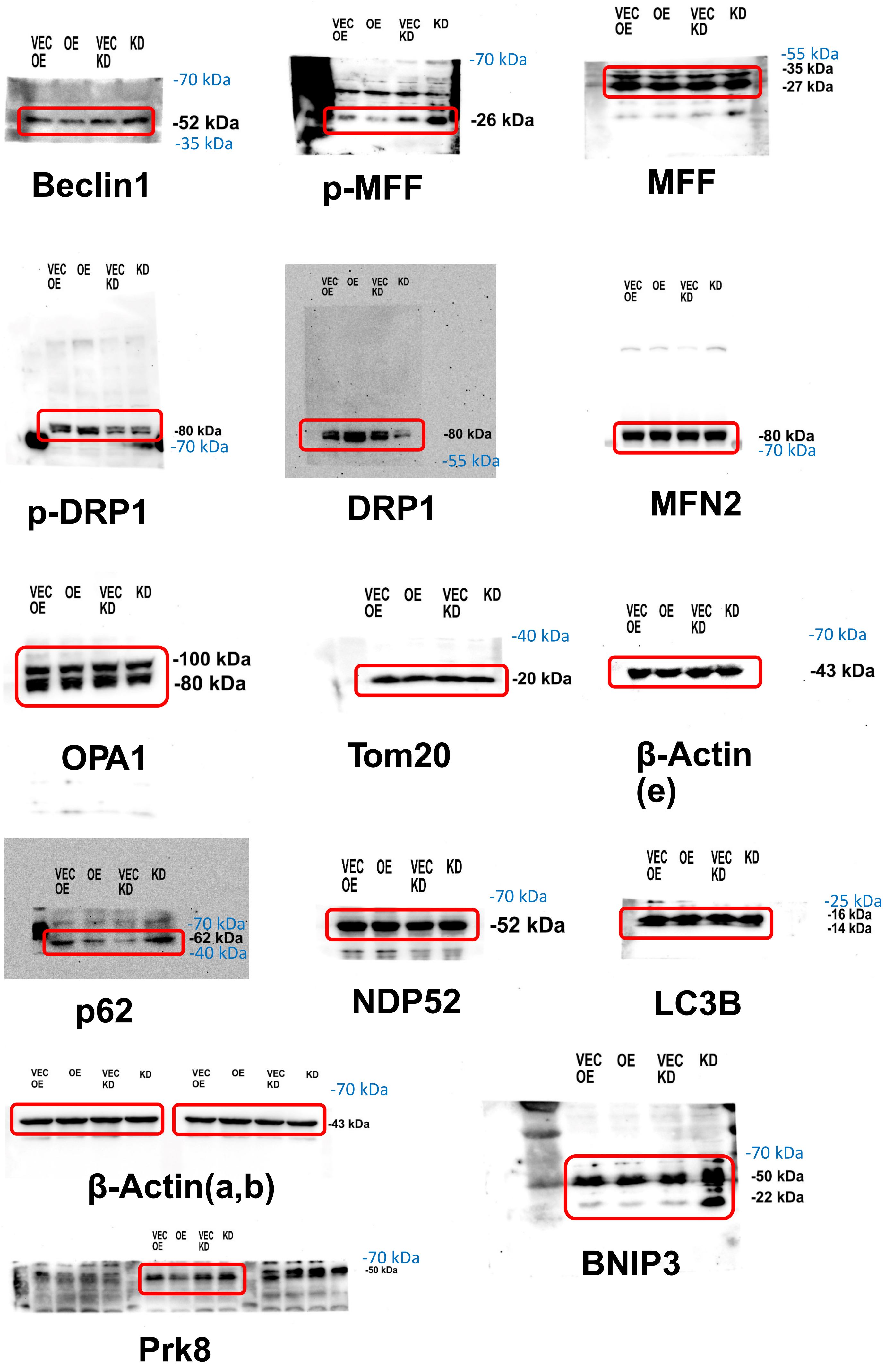
VEC OE OE VEC KD KD
-70 kDa
-52 kDa
-35 kDa
Beclin1
VEC OE OE VEC KD KD
-70 kDa
-26 kDa
p-MFF
VEC OE OE VEC KD KD
-55 kDa
-35 kDa
-27 kDa
MFF
VEC OE OE VEC KD KD
-80 kDa
-70 kDa
p-DRP1
VEC OE OE VEC KD KD
-80 kDa
-55 kDa
DRP1
VEC OE OE VEC KD KD
-80 kDa
-70 kDa
MFN2
VEC OE OE VEC KD KD
-100 kDa
-80 kDa
OPA1
VEC OE OE VEC KD KD
-40 kDa
-20 kDa
Tom20
VEC OE OE VEC KD KD
-70 kDa
-43 kDa
β-Actin (e)
VEC OE OE VEC KD KD
-70 kDa
-62 kDa
-40 kDa
p62
VEC OE OE VEC KD KD
-70 kDa
-52 kDa
NDP52
VEC OE OE VEC KD KD
-25 kDa
-16 kDa
-14 kDa
LC3B
VEC OE OE VEC KD KD
VEC OE OE VEC KD KD
-70 kDa
-43 kDa
β-Actin(a,b)
VEC OE OE VEC KD KD
-70 kDa
-50 kDa
-22 kDa
BNIP3
VEC OE OE VEC KD KD
-70 kDa
-50 kDa
Prk8

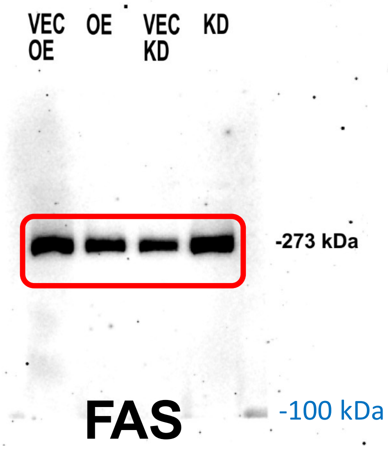

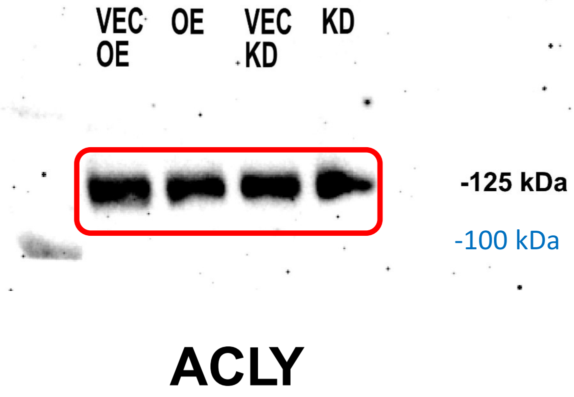

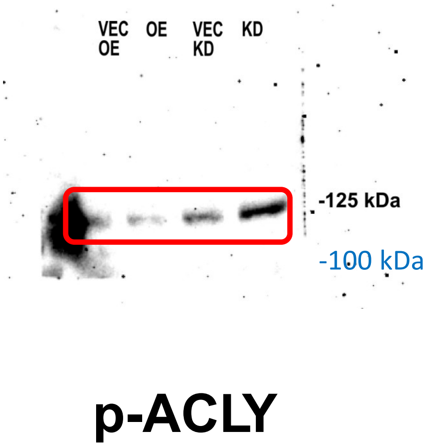

Please replace “hyphen” with “minus” in Figure

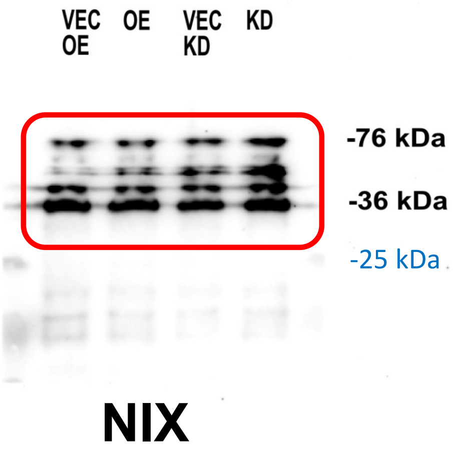

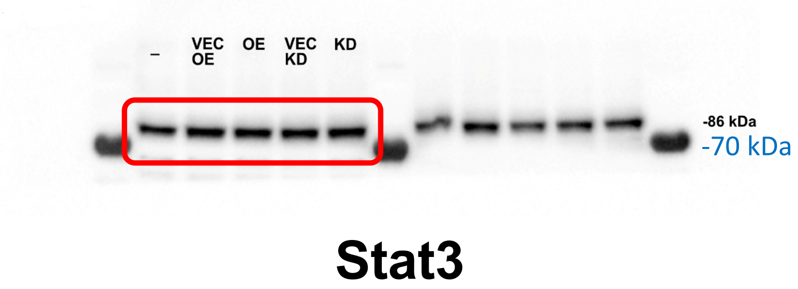

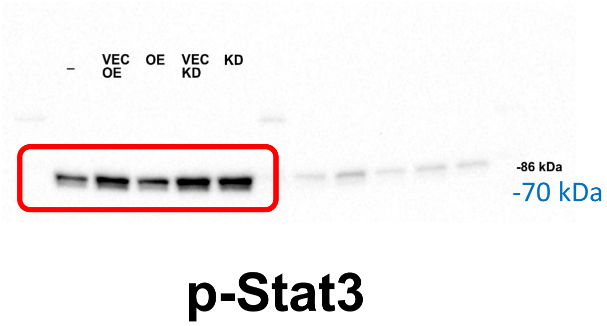

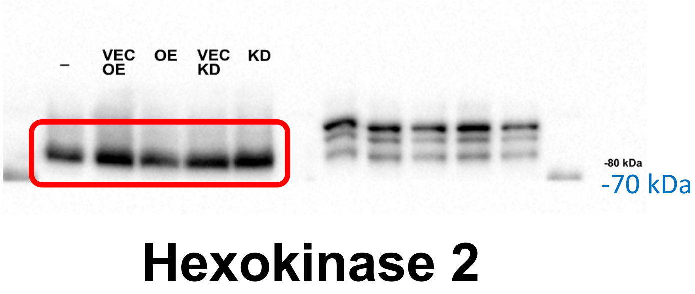

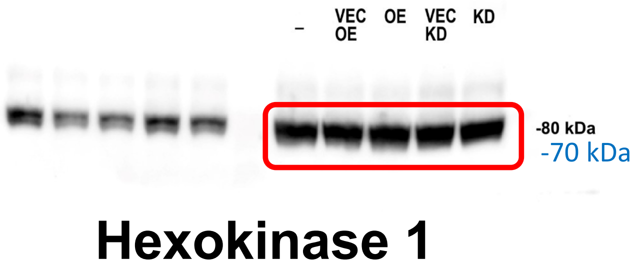

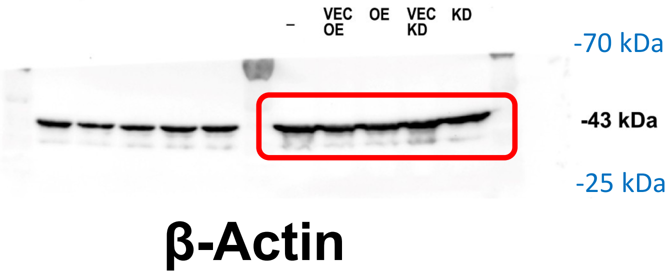

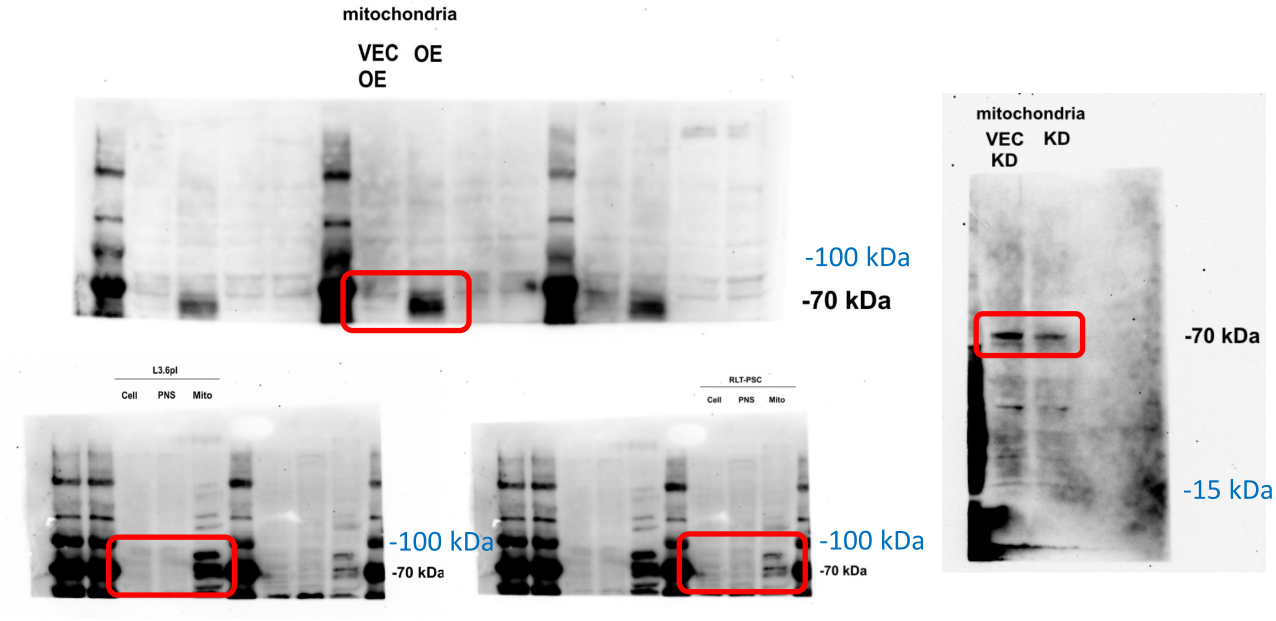

IL-17RB

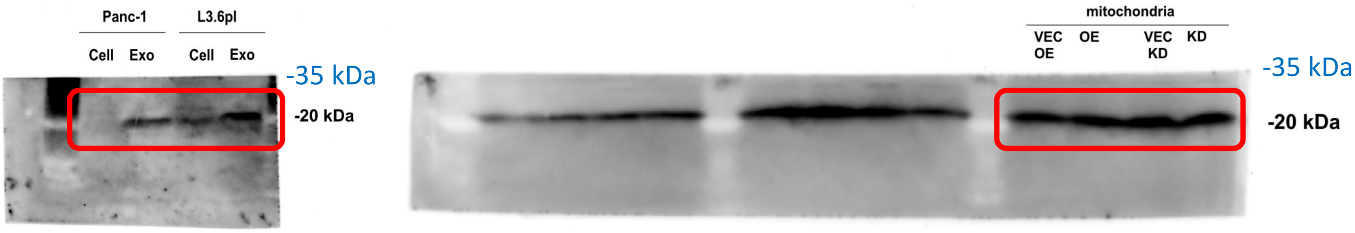

IL-17B

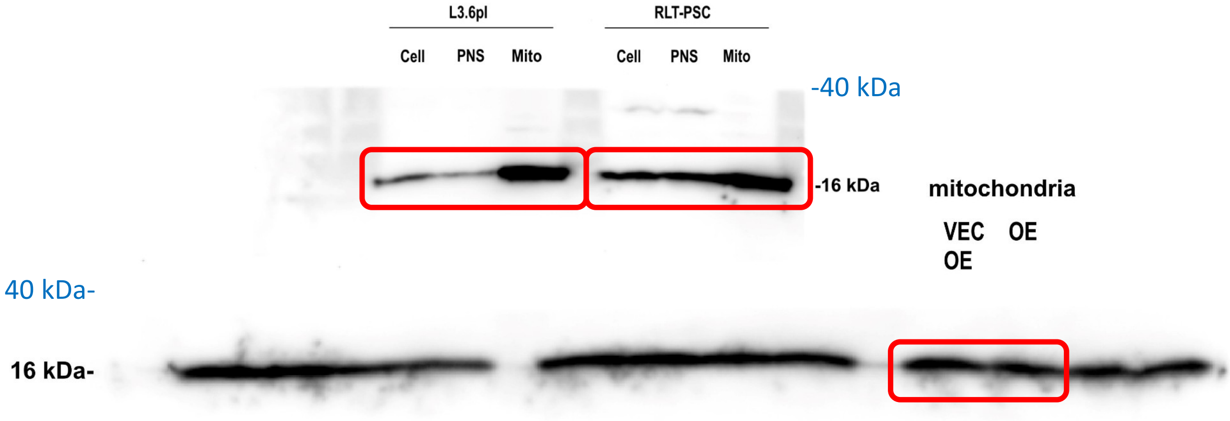

Tom20

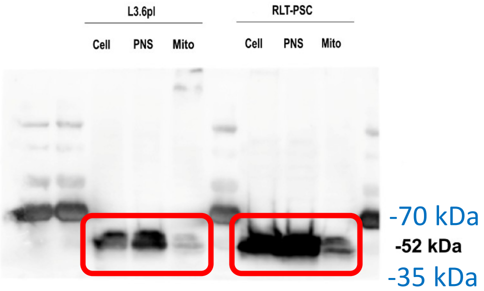

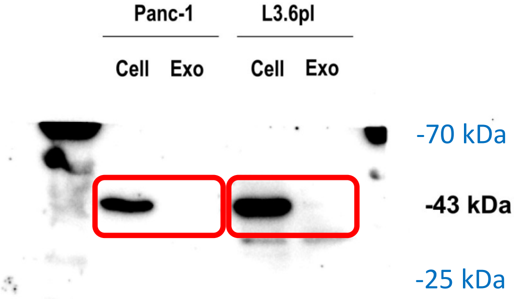

α-Tublin **β-Actin**

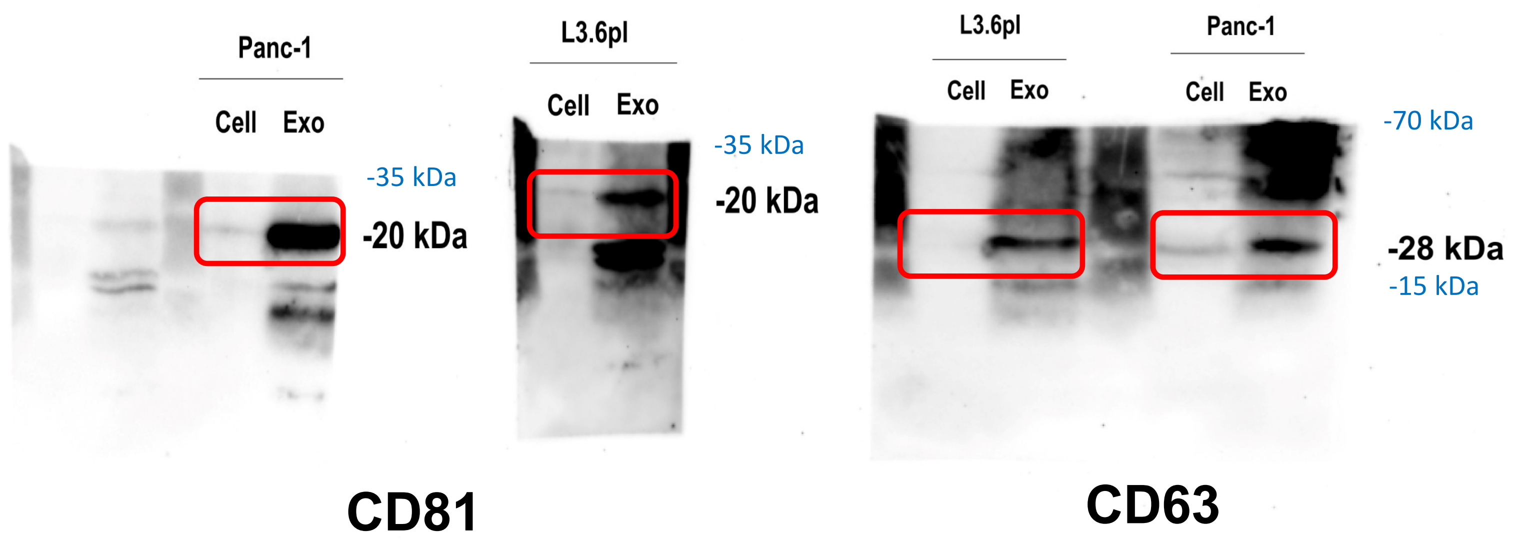
Panc-1
Cell Exo
-35 kDa
-20 kDa
L3.6pl
Cell Exo
-35 kDa
-20 kDa
L3.6pl
Cell Exo
Panc-1
Cell Exo
-70 kDa
-28 kDa
-15 kDa
CD81
CD63

Figure 7.

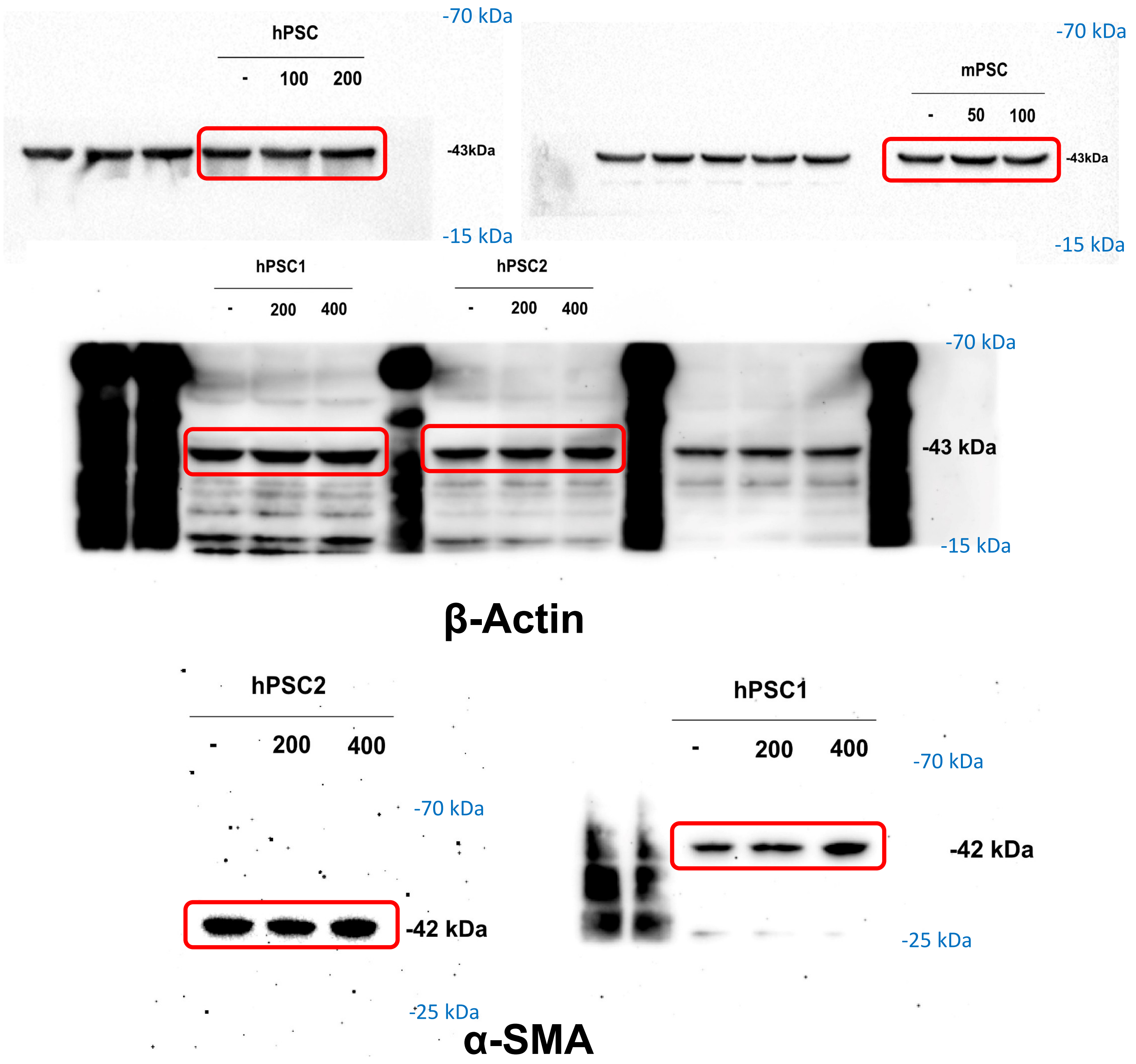
hPSC
- 100 200
-70 kDa
-43kDa
-15 kDa
mPSC
- 50 100
-70 kDa
-43kDa
-15 kDa
hPSC1
- 200 400
hPSC2
- 200 400
-70 kDa
-43 kDa
-15 kDa
β-Actin
hPSC2
- 200 400
-70 kDa
-42 kDa
-25 kDa
hPSC1
- 200 400
-70 kDa
-42 kDa
-25 kDa
α-SMA

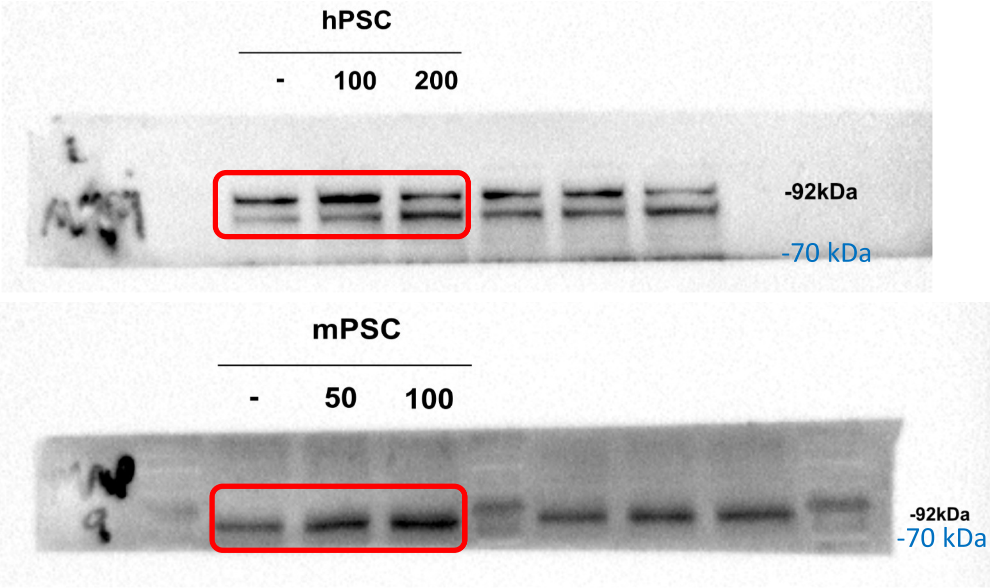

MMP9

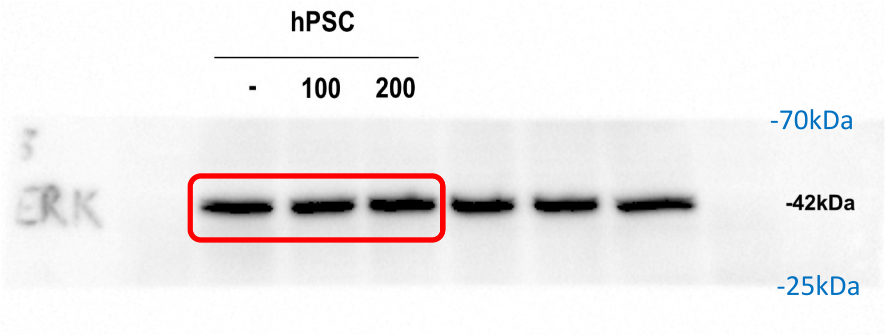

ERK1/2

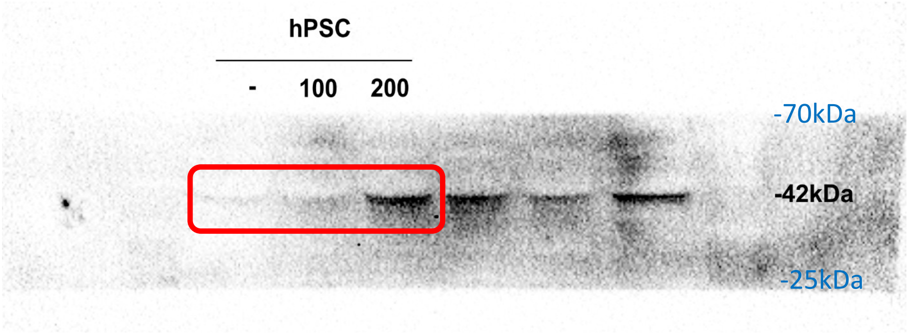

p-ERK1/2

Figure S2. Quantification of the Western blots.
