# Supplementary material for: IL-17B/RB Activation in Pancreatic Stellate Cells Promotes Pancreatic Cancer Metabolism and Growth
Source: Cancers (Basel). 2021 Oct 24;13(21):5338. doi: 10.3390/cancers13215338 (PMC8611647; doi:10.3390/cancers13215338)
Supplement: Supplementary file 1 [file cancers-13-05338-s001.zip › cancers-1330593-Supplementary Figure S1.pdf]

A

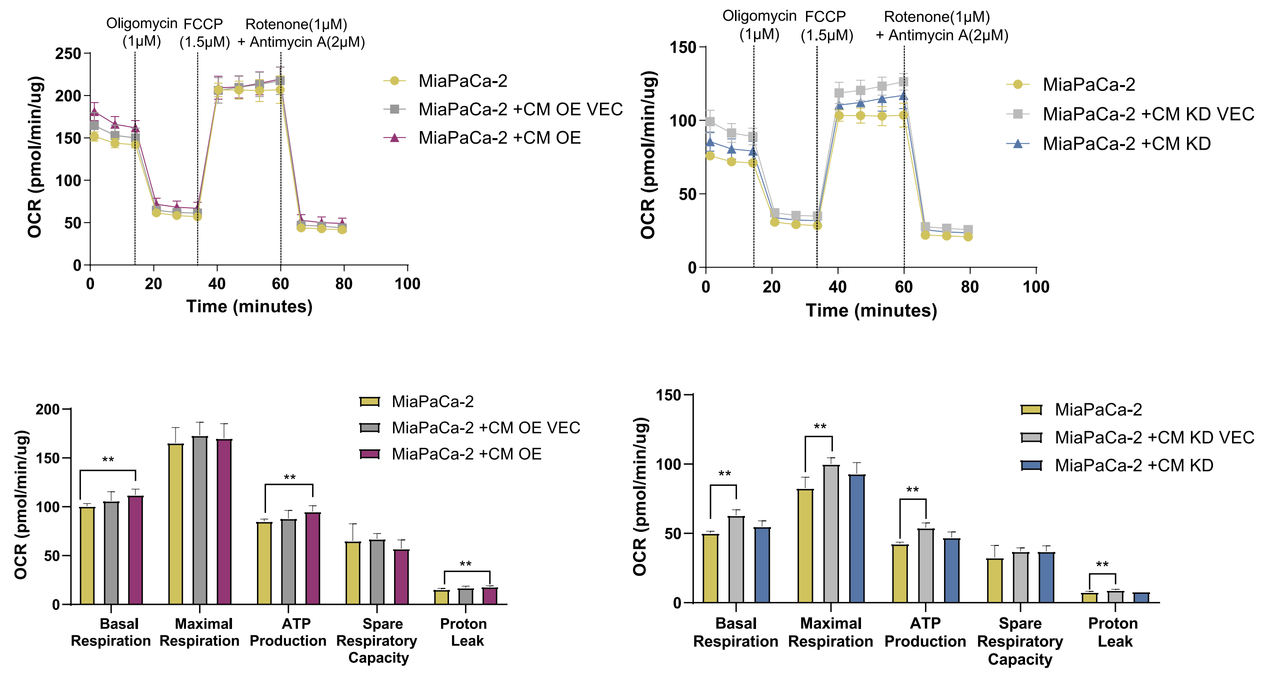
Oligomycin (1μM)
FCCP (1.5μM)
Rotenone(1μM) + Antimycin A(2μM)
OCR (pmol/min/ug)
Time (minutes)
MiaPaCa-2
MiaPaCa-2 +CM OE VEC
MiaPaCa-2 +CM OE
MiaPaCa-2 +CM KD VEC
MiaPaCa-2 +CM KD
Basal Respiration
Maximal Respiration
ATP Production
Spare Respiratory Capacity
Proton Leak
**

B

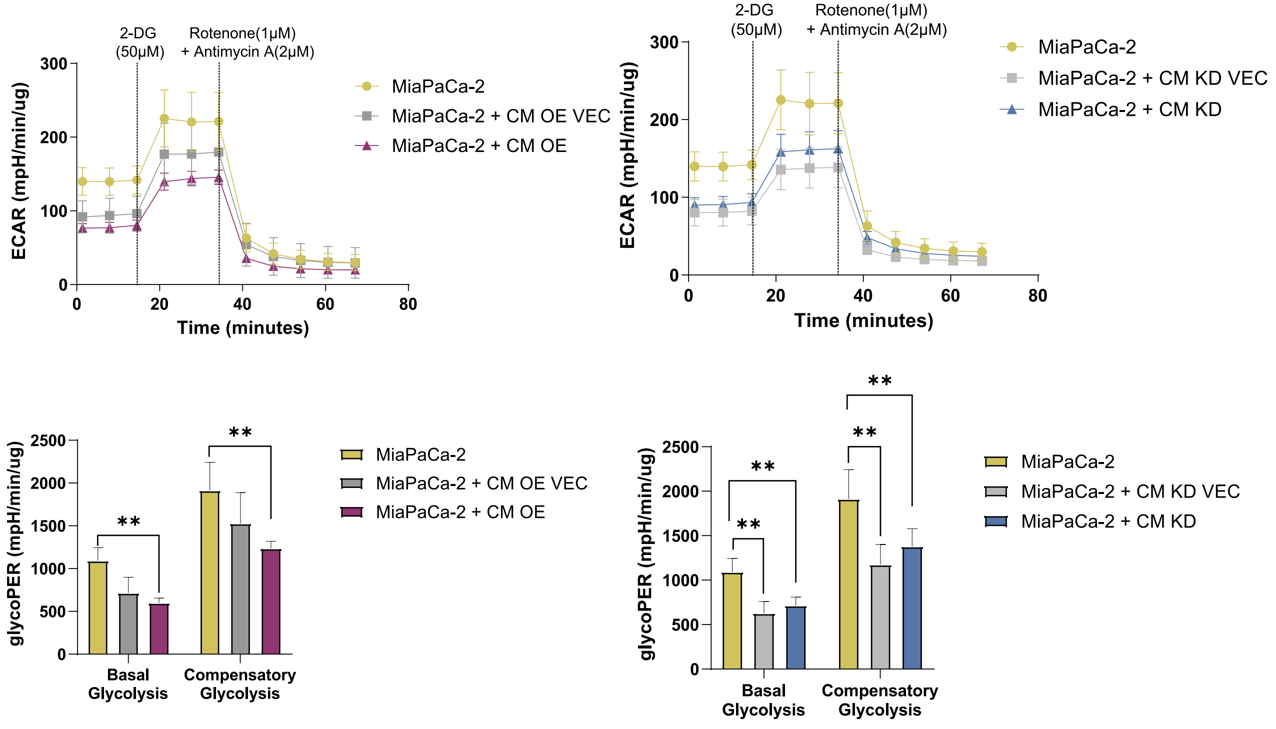
2-DG (50μM)
Rotenone(1μM) + Antimycin A(2μM)
ECAR (mpH/min/ug)
Time (minutes)
MiaPaCa-2
MiaPaCa-2 + CM OE VEC
MiaPaCa-2 + CM OE
MiaPaCa-2 + CM KD VEC
MiaPaCa-2 + CM KD
glycoPER (mpH/min/ug)
Basal Glycolysis
Compensatory Glycolysis
**

Figure S1. IL-17RB overexpressing PSCs increase mitochondrial respiration and decrease glycolysis - same experiments as in Figure 5 with Mia PaCa-2 tumor cells.
